# Editorial: Micronutrients movement from soil to the grains: Role of plant membrane transporters

**DOI:** 10.3389/fpls.2023.1179674

**Published:** 2023-03-22

**Authors:** Jing Che, Felipe Klein Ricachenevsky, Fenglin Deng

**Affiliations:** ^1^ State Key Laboratory of Soil and Sustainable Agriculture, Institute of Soil Science, Chinese Academy of Sciences, Nanjing, China; ^2^ University of Chinese Academy of Sciences, Beijing, China; ^3^ Graduate Program in Cellular and Molecular Biology, Center of Biotechnology, Institute of Biosciences, Federal University of Rio Grande do Sul, Porto Alegre, Brazil; ^4^ Graduate Program in Botany, Botany Department, Institute of Biosciences, Federal University of Rio Grande do Sul, Porto Alegre, Brazil; ^5^ MARA Key Laboratory of Sustainable Crop Production in the Middle Reaches of the Yangtze River (Co-construction by Ministry and Province), College of Agriculture, Yangtze University, Jingzhou, China

**Keywords:** biofortification, membrane transporter, movement from soil to grain, micronutrient acquisition, function characterization

Micronutrients are required in smaller amounts than macronutrients for their growth (Hansch and Mendel, 2009). Plant micronutrients include iron (Fe), manganese (Mn), copper (Cu), zinc (Zn), boron (B), molybdenum (Mo), chloride (Cl), and nickel (Ni). These micronutrients are first taken up by plant roots from the soil, and their availability varies greatly depending on soil conditions. For example, when soil pH falls by one unit, the concentration of Zn in the soil solution increases 100 times (Rengel, 2015). To cope with such soil constraints, plants have evolved strictly regulated mechanisms of nutrient acquisition. Among them, plant transporters play an important role in maintaining ion homeostasis by controlling the movement of ions across the membrane (Vishwakarma et al., 2019; Huang et al., 2020; Ma and Tsay, 2021).

After taken up by the roots, micronutrients are then translocated to the shoots, distributed to various organs and tissues, and finally delivered to the grains. As micronutrients in the soil, the imbalance of micronutrients in plants can negatively impact plant growth. For example, Mn deficiency causes interveinal chlorosis in young expanded leaf blades, while excess Mn causes brown spots on the mature leaves (Hannam and Ohki, 1988). Plants use a variety of transporters to maintain basic cellular processes by facilitating the movement of ions across membranes. Several transporters involved in the movement of micronutrients from the soil to grains have been identified, with many more to be discovered in the future. Moreover, most of the transporters identified to date come from model plants such as rice, while the function of transporters in other crops such as wheat and maize is largely unknown.

In this Research Topic, we include a review article and three research articles covering aspects of transporter identification, characterization of transporter function and utilization of transporters for biofortification. Eleven *TcZIP* genes have been identified in cacao (*Theobroma cacao* L.) based on a homology search of the *Arabidopsis thaliana* zinc/iron-regulated transporter-like proteins (ZIP), which are involved in the uptake of metal ions (such as Fe and Zn) (Pacheco et al.). This study analyzed the physicochemical properties, three-dimensional protein structure, and promoter region of *ZIP* genes in cacao, which expanded the knowledge of ZIP genes from model plants to the economically relevant plant cacao, but the function of these genes in planta remains to be characterized.

Unlike Fe and Zn, which are required by both humans and plants, selenium (Se) is required only by humans. It is still unclear whether Se is essential for plants or not. In this collection, eight transporters have been identified in Se uptake in proso millet (*Panicum miliaceum* L.). Data on expression of these transporters suggest that they play an important role in salt stress mitigation (Mushtaq et al.). The function and physiological roles of these genes should be characterized by investigating their subcellular and tissue localization, gene expression patterns, and phenotypic analysis using knockout or overexpression lines.

Understanding the molecular mechanisms underlying the transport of micronutrients from soil to grains is critical for the accumulation of micronutrients in edible parts. Hu et al. characterized the function of a molybdate transporter, OsMOT1;2 in rice. They found that OsMOT1;2 is involved in the delivery of Mo to grains by regulating the translocation of Mo from root-to-shoot and the remobilization of Mo from leaves to grains. Overexpression of *OsMOT1;2* increased the accumulation of Mo in rice grains, which could benefit human nutritional needs and improve adaptation to low Mo soils.

Some of the identified micronutrient transporters have been used to enhance the biofortification of micronutrients. Wairich et al. summarized the approaches to increase Fe/Zn in rice grains, which included the manipulation of Fe/Zn-related transporters. In comparison with the fundamental understanding of Fe transport from soil to grain, knowledge of the molecular mechanisms of other micronutrients transport to grain is still insufficient. More transporters are expected to be characterized and manipulated in the future to improve plant adaptation to soil stresses and generate micronutrient biofortified plants using both conventional and transgenic approaches.

## Author contributions

All authors listed have made a substantial, direct and intellectual contribution to the work and approved it for publication.
